# Learning Preference Profiles and Visual Narratives in Higher Education: Relevance to Human Intelligence Research

**DOI:** 10.3390/jintelligence14100250

**Published:** 2026-10-09

**Authors:** Sebastián David Bohórquez-Ponce, Efraín de Jesús Hernández Buelvas, Erika María López-López, Osnamir Elias Bru-Cordero, Cristian David Correa-Álvarez

**Affiliations:** 1 Dirección Académica, Universidad Nacional de Colombia, Sede de La Paz, Kilómetro 9 Vía Valledupar-La Paz, Valledupar 202017, Colombia; sebohorquezp@unal.edu.co (S.D.B.-P.); erlopezl@unal.edu.co (E.M.L.-L.); oebruc@unal.edu.co (O.E.B.-C.); 2 Facultad de Ciencias Humanas, Arte y Diseño, Universidad del Sinú–Elías Bechara Zainúm, Montería 230003, Colombia; efrainhernandezb@unisinu.edu.co; 3 Departamento de Matemáticas y Estadística, Universidad Nacional de Colombia, Sede Manizales, Kilómetro 7 Vía al Aeropuerto, Manizales 170003, Colombia

**Keywords:** learning preference profiles, CHAEA, visual narratives, mixed methods, metacognition, higher education, human intelligence

## Abstract

University students differ in how they approach unfamiliar information, yet learning-style labels provide a weak basis for matching instruction to individuals. This study examined learning preference profiles and visual narratives in higher education, considering their relevance and limits for human intelligence research. A convergent mixed-methods secondary analysis included 144 complete questionnaire records and 12 interpretable drawings at a Colombian public university. The adapted 80-item instrument used five response categories, with Frequently and Always counted as endorsements. Reflective scores were highest, followed by Theoretical, Pragmatic, and Active scores ($\chi^{2}\left(3\right)=158.27$, p<0.001; Kendall’s W=0.366). The analytic composite exceeded the action-oriented composite ($r_{rb}=0.891$, p<0.001). This ordering persisted across four scoring representations, although effect sizes and tied maxima varied. Drawings mainly represented learning through direction, staged progress, obstacles, and internal processing. Missing participant explanations, incomplete coding records, and absent participant-level linkage restricted integration to exploratory descriptive comparison. The study offers methodological considerations for human intelligence research by distinguishing descriptive patterns from claims about cognitive capacities. Neither intelligence nor metacognitive performance was assessed, and instructional implications remain hypotheses requiring prospective evaluation.

## 1. Introduction

Research on human intelligence provides a broader context for questions about how people approach cognitively demanding learning tasks. Related work on metacognition and self-regulated learning examines planning, monitoring, and adaptation of strategies (Dörrenbächer-Ulrich et al., 2024), while longitudinal evidence links metacognition with subsequent academic achievement (He et al., 2024). In the present study, this literature provides theoretical context rather than a set of measured outcomes. The empirical analysis concerns responses to a learning-preference questionnaire and visual representations of learning; neither source provides a direct measure of intelligence or metacognitive performance.

University classrooms are cognitively, culturally, and academically heterogeneous. Students enter the same course with different prior knowledge, habits of study, levels of confidence, preferred ways of approaching uncertainty, and resources for turning information into usable understanding. That diversity is especially visible at regional campuses that bring together students from urban and rural communities, multiple admission pathways, and disciplines with markedly different epistemic traditions. For instructors, the practical question is not whether students differ; it is how to design a common learning environment that gives those differences room to operate without reducing learners to fixed categories (Shane-Simpson et al., 2025).

Learning-style frameworks have long offered an intuitively attractive answer. They promise a language for describing how students prefer to act, reflect, conceptualize, or apply what they encounter. Yet the strongest version of that promise—that learning improves when instruction is matched to a person’s diagnosed style—has not held up well under controlled testing. Pashler et al. (2008) argued that evidence for the required aptitude-by-treatment interaction was missing, and subsequent work has continued to identify conceptual ambiguity, limited validation, and the risk of turning flexible preferences into identity-like labels (Cuevas, 2015; Kirschner, 2017; Newton, 2015). More recently, Hattie and O’Leary (2025) distinguished learning styles from preferences and strategies, helping explain why the language of styles persists even when the matching hypothesis is rejected. Empirical studies in university settings likewise indicate that reported preferences do not reliably predict which instructional medium will produce stronger comprehension (Husmann & O’Loughlin, 2019; Rogowsky et al., 2015).

Rejecting style matching does not require discarding every descriptive question that style inventories raise. A classroom profile can still prompt useful reflection when it is treated as a provisional account of how a group reports approaching learning rather than as a prescription for individualized teaching (Duff & Duffy, 2002; Hattie & O’Leary, 2025). A high Reflective score, for example, may indicate that many students report valuing time to examine evidence before responding; it does not mean that they should be shielded from discussion, experimentation, or action. In this study, the Honey–Alonso Learning Styles Questionnaire (CHAEA) is therefore used as a descriptive preference inventory. Its scores are interpreted as overlapping, cohort-level learning preference profiles rather than as stable cognitive types, measures of intellectual ability, or prescriptions for style-matched instruction.

A second line of inquiry begins from a different premise: people do not only report learning through questionnaire items; they also represent it through stories, images, spatial arrangements, metaphors, and omissions. Bruner (1991) described narrative as a mode of organizing experience into meaningful relations rather than a decorative way of presenting information. In educational settings, multimodal meaning-making can make visible forms of understanding that may not appear in conventional prose (Cohn, 2013; Jewitt, 2008). A path, staircase, spiral, barrier, or diagram of a mind at work can condense a learner’s sense of sequence, difficulty, agency, and purpose. Ainsworth et al. (2011) showed why drawing matters for learning: producing an image can externalize relations and support the organization of ideas. Research on drawing-to-learn and visual literacy also cautions that images are not transparent windows into cognition; their meaning depends on task, context, representational conventions, and interpretation (Farrar et al., 2024; Quillin & Thomas, 2015).

The relationship between preference profiles and visual narratives is therefore potentially productive, but only if one source is not forced to “validate” the other. Questionnaire scores summarize patterned self-reports across many items, whereas drawings preserve ambiguity, selectivity, and spatial organization. Their points of contact may illuminate different aspects of how a cohort reports and represents learning, while their differences can expose what one method leaves out. Johnson et al. (2007) positioned mixed methods as an approach for drawing on the complementary strengths of quantitative and qualitative inquiry. In convergent designs, integration requires an explicit comparison of where findings converge, diverge, or address different layers of a phenomenon (Fetters et al., 2013; Guetterman et al., 2015). In the present study, however, the two strands differ substantially in evidentiary weight: the quantitative analysis includes 144 complete questionnaire records, whereas the retained qualitative corpus contains only 12 interpretable drawings that cannot be linked reliably to individual questionnaire records. Consequently, integration is restricted to cohort-level comparison and cannot establish associations between particular visual motifs and particular preference profiles.

This integration is relevant to contemporary higher education, where learning environments increasingly combine text, visualization, video, simulation, conversational systems, and student-generated media. Rapidly expanding educational technologies reinforce the need for rigorous, ethical, and learner-centered evidence rather than novelty-driven adoption (Bond et al., 2024). The same principle applies to multimodality: offering several representational routes may widen participation, but it does not justify assigning a preferred channel to each learner (González-Afonso et al., 2026). Current debates surrounding generative artificial intelligence further highlight the importance of representational flexibility, authorship, interpretation, and equitable access (Bond et al., 2024; Yan et al., 2024).

The present study was conducted at the Universidad Nacional de Colombia, Sede de La Paz, a regional campus serving students across six undergraduate programs. An adapted CHAEA instrument was administered to 144 students, and a purposive subgroup was invited to produce written and visual accounts of perceived learning. The quantitative dataset contained 144 complete questionnaire records, whereas the audited qualitative documentation retained 12 interpretable drawings. Participant explanations and reliable case-level links between the visual and questionnaire records were not available; accordingly, the visual evidence is interpreted only within the boundaries of the retained corpus. Written responses that were blank, illegible, or incompletely preserved are not treated as evidence of a visual or multimodal learning preference.

The study addresses one overarching question: How are CHAEA-derived learning preference profiles distributed within this student cohort, and what does the retained visual corpus contribute to a cohort-level understanding of how learning is represented? Its objective is to characterize the four reported preference dimensions, examine the reliability and robustness of their observed differences, describe recurrent formal features in the available drawings, and integrate the two strands at the level supported by the preserved evidence. The analysis concerns reported learning preferences and visible representational features, not individual levels of metacognition, self-regulation, or intellectual ability.

The study contributes a within-cohort analysis of CHAEA-derived score distributions and their sensitivity to alternative scoring rules, accompanied by a descriptive account of the retained visual material. Its relevance to human intelligence research concerns how learning-related self-reports and visual representations are interpreted in relation to cognitive and regulatory constructs. The analysis distinguishes the patterns observable in these sources from claims that would require direct measures of cognitive performance, metacognitive monitoring, and linked participant data. It therefore provides descriptive findings and methodological considerations for further research, without treating preferences or visual motifs as measures of intelligence.

## 2. Conceptual and Empirical Framework

This section brings together four bodies of work that are often discussed separately: learning preference inventories, narrative and multimodal representation, metacognition and self-regulation, and mixed-methods integration. The point of contact is not an assumption that a drawing can reveal a student’s true style. Rather, each tradition offers a different way to examine how learners orient themselves toward information, action, reflection, and meaning. Their combination supports an interpretation that is descriptive, situated, and pedagogically cautious.

### 2.1. Metacognition, Self-Regulation, and Process-Oriented Intelligence Research

Metacognition and self-regulated learning provide a theoretical context for interpreting learning-related self-reports, but they were not directly assessed in this study. Metacognition concerns learners’ knowledge of persons, tasks, and strategies, together with their capacity to plan, monitor, and evaluate cognitive activity (Dörrenbächer-Ulrich et al., 2024; Pintrich, 2002). Zimmerman (2002) situated these processes within cyclical patterns of forethought, performance, monitoring, and self-reflection, while later reviews have reinforced their central role in self-regulated learning (Panadero, 2017). Longitudinal evidence associates metacognition with subsequent academic achievement (He et al., 2024); it does not establish that learning-preference scores or visual motifs can serve as substitutes for measures of that construct.

Self-reports about learning are necessarily partial. A student can describe a preference for careful observation without monitoring effectively in a real task, and a student who values action may still plan strategically. Beliefs about learning also reflect disciplinary cultures, institutional expectations, and prior experiences with assessment and support (Lawson et al., 2019). The questionnaire profiles and visual representations are therefore treated as distinct descriptive sources. Neither establishes how accurately a participant monitored understanding or how effectively that participant regulated learning.

### 2.2. From Learning Styles to Learning Preference Profiles

The Honey–Mumford tradition and its Spanish-language adaptation in CHAEA describe four positions in an experiential sequence: Active, Reflective, Theoretical, and Pragmatic. Active items emphasize novelty, participation, and immediate involvement; Reflective items emphasize observation, information gathering, caution, and consideration of alternatives; Theoretical items emphasize logic, coherence, structure, and underlying principles; and Pragmatic items emphasize usefulness, application, and testing ideas in practice. Although these dimensions are often converted into a dominant “style,” the underlying scores are better interpreted as overlapping self-reported preferences that may vary with task, experience, and context (Kolb & Kolb, 2005).

Duff and Duffy (2002) found that psychometric examination of the Honey–Mumford questionnaire raised questions about its structure and the interpretation of its scales. That concern is relevant to CHAEA because adaptation across languages, populations, and response formats can alter the meaning and measurement properties of an instrument. Experiential models nevertheless remain attractive because they describe recognizable learning actions and can prompt instructors to consider whether a course overuses one part of the learning cycle (Kanadli, 2016). Used in this limited way, a profile can serve as a descriptive account of a classroom rather than as a diagnostic endpoint.

The more consequential limitation concerns instructional matching. Pashler et al. (2008) specified the type of evidence required to demonstrate that learners benefit when instruction is matched to a diagnosed style, but most studies have not met that standard. Direct empirical tests likewise have not supported the claim that matching auditory or visual preferences to instructional mode improves comprehension (Rogowsky et al., 2015). Subsequent critiques have emphasized the conceptual and educational risks of turning flexible preferences into fixed learner identities (Kirschner, 2017; Newton, 2015; Newton & Miah, 2017). More recently, Hattie and O’Leary (2025) distinguished preferences and learning strategies from stable learner types, reinforcing a more cautious interpretation of reported differences.

This distinction changes the pedagogical question. Instead of asking how a “Reflective learner” should be taught, instructors can ask whether a course offers sufficient opportunities for all students to inspect evidence, form conceptual models, test ideas, receive feedback, and revise their reasoning. Effective learning strategies such as retrieval, elaboration, distributed practice, and self-explanation are not tied to declared learning styles (Dunlosky et al., 2013; Freeman et al., 2014). Variety is therefore justified by the demands of learning and equitable participation rather than by the assumption that each student possesses a single optimal instructional channel.

In the present study, the four CHAEA-derived scores are consequently treated as a within-person preference profile observed at one point in time. A higher score indicates more frequent endorsement of statements associated with that dimension under the administered response format. It does not indicate greater intelligence, superior learning, or a stable cognitive capacity. The profile terminology is retained to preserve continuity with the source instrument, while its interpretation is shifted from categorical classification toward relative orientation.

### 2.3. Visual Narrative and Multimodal Meaning-Making

Narrative provides a complementary perspective because learning is experienced not only as a set of reported preferences but also as a temporal and interpretive process. Bruner (1991) argued that narrative organizes experience through intention, sequence, expectation, and consequence. Educational narratives can therefore position learners in relation to challenge, agency, others, and imagined destinations (Murphy, 2024; Polkinghorne, 1995). Visual narratives do not require a complete written plot; spatial arrangements can imply beginnings, transitions, obstacles, cycles, and goals (Cohn, 2013).

Images are not simply abbreviated sentences. Visual arrangements distribute attention through position, size, direction, enclosure, connection, repetition, and contrast (Cohn, 2013; Jewitt, 2008). A staircase can make progression visible, a maze can materialize uncertainty and choice, and a diagram connecting information to a head can foreground internal cognitive processing. Such interpretations should remain anchored in recurrent formal features and task context rather than in projective claims about personality or cognitive traits.

Ainsworth et al. (2011) described drawing as a generative activity that can require learners to select, organize, and integrate information. Drawings may externalize relationships that students consider relevant, although their meaning depends on the prompt, representational conventions, and opportunities to explain or revise the image (Farrar et al., 2024; Quillin & Thomas, 2015). Digital storytelling broadens these possibilities through combinations of image, voice, written language, music, and editing (Robin, 2008; Sadik, 2008). Experimental and applied studies have associated carefully designed storytelling activities with critical thinking, motivation, retention, and knowledge transfer under particular instructional conditions (Ginting et al., 2024; Yang & Wu, 2012). Such evidence concerns designed learning activities, however, and does not imply that spontaneous drawings reveal stable modality preferences.

Visual expression may also broaden participation when conventional academic language does not fully capture experience. Research on visual literacy emphasizes that modes distribute meaning differently and require distinct interpretive competencies (Farrar et al., 2024). Recent work examining spatial, geometric, sensory, and affective forms of expression similarly challenges narrow assumptions about legitimate educational representation (Thom & Weber, 2026). The relevant implication is representational plurality rather than a new basis for labeling learners.

### 2.4. Mixed-Methods Integration and Evidentiary Boundaries

The relationship between preference profiles and visual narratives is potentially informative only if one source is not forced to validate the other. Questionnaire scores summarize patterned responses across many items, whereas drawings preserve ambiguity, selectivity, and spatial organization. Mixed-methods research provides a framework for comparing these different forms of evidence while preserving their distinct contributions (Fetters et al., 2013; Johnson et al., 2007).

Fetters et al. (2013) explained that integration can occur through several explicit strategies, including merging results and comparing complementary forms of evidence. Joint displays can make these relationships visible by placing quantitative patterns alongside qualitative themes without collapsing one form of evidence into the other (Guetterman et al., 2015). Nevertheless, integration does not remove differences in sample size, preservation, unit of analysis, or inferential strength.

These evidentiary differences are particularly important in the present study. The quantitative strand comprises 144 complete questionnaire records, whereas the retained qualitative corpus contains only 12 interpretable drawings from a planned activity involving 30 invitees. Participant explanations, the complete raw visual corpus, and case-level coding records were not preserved, and the drawings could not be linked reliably to individual questionnaire records. The visual strand is therefore interpreted only as a descriptive account of the available material, while mixed-methods integration is restricted to cohort-level comparison. No participant-level association between a preference profile and a particular visual motif is inferred.

## 3. Materials and Methods

This section describes the study design, institutional setting, participants, data sources, scoring procedures, quantitative and qualitative analyses, mixed-methods integration, and ethical safeguards. The quantitative and visual strands are presented separately before describing their cohort-level integration.

The study used a convergent mixed-methods design for the secondary analysis of questionnaire and visual materials. The sources were analyzed separately and subsequently brought together in a joint display. The quantitative analyses provided the principal empirical results, while the incomplete visual material provided descriptive context. Cross-source comparisons were exploratory rather than confirmatory. The questionnaire item allocation, complete exploratory demographic results, and scoring-rule sensitivity analyses are provided in Appendix A.

### 3.1. Setting and Study Context

The research was conducted at the Universidad Nacional de Colombia, Sede de La Paz, in the department of Cesar. The campus offers undergraduate programs that span quantitative, engineering, biological, social, territorial, and cultural fields. At the time of data collection, the six represented programs were Statistics, Mechatronics Engineering, Biology, Biological Engineering, Geography, and Cultural and Communication Management. This disciplinary range made the site appropriate for examining group-level variation without assuming that one academic culture represented the entire campus.

Survey records were collected between 2 May and 8 July 2025 through an institutional online form. The source workbook contained a timestamp, consent response, contact and identification fields, demographic variables, and 80 learning-preference items. Direct identifiers were used only to audit duplicates and completeness; they were removed before analysis and are not included in the files prepared for reporting or repository deposition.

Participation was voluntary. The preserved documentation does not include the total number of students who received the invitation or a denominator from which a response rate can be calculated. The quantitative sample is therefore treated as a convenience sample comprising the complete set of usable records available for this secondary analysis, rather than as a probability sample of the campus or Colombian university students.

### 3.2. Participants and Available Data Sources

The quantitative dataset contained 144 records. Every record indicated consent, included responses to all 80 preference items, and had complete values for the principal demographic variables used in this analysis. No duplicate email addresses or identification numbers were detected. Because the dataset represented the complete set of usable responses supplied for secondary analysis, no prospective power calculation or additional sampling was undertaken. Inferential statistics are interpreted as estimates of patterns within this cohort rather than evidence that the sample is representative of Colombian university students.

The original qualitative activity planned to involve 30 students, with five students from each academic program, who were invited to provide an autobiographical text and a drawing about their learning. The preserved materials contained 12 drawings that were sufficiently interpretable for visual analysis. Participant explanations, the complete case-level coding history, and a reliable key linking drawings to questionnaire records were not available. Qualitative claims are therefore restricted to visible features of these 12 drawings. Blank, illegible, or incompletely preserved written responses were not interpreted as evidence of visual preference, reluctance to write, literacy, or any other psychological characteristic.

### 3.3. Instrument and Score Construction

The questionnaire comprised 80 Spanish-language items derived from the CHAEA framework, with 20 items assigned to each of four profiles: Active, Reflective, Theoretical, and Pragmatic. The item allocation is provided in Appendix A.1. Unlike the original dichotomous response format, the administered form asked students to respond on a five-point frequency scale: 1 (Never), 2 (Rarely), 3 (Sometimes), 4 (Frequently), and 5 (Always). The use of a five-category response scale constitutes an adaptation, and the resulting scores should not be assumed to inherit the original instrument’s norms or validity evidence.

Two representations of the item responses were retained. First, the five-category values were kept to examine item-level internal consistency without discarding response variation. Second, the scoring rule documented in the source analysis was reproduced: responses of 4 or 5 were coded 1, and responses of 1–3 were coded 0. Binary items were summed within each profile, yielding scores from 0 to 20.

The 4–5 threshold was retained because Frequently and Always represent sustained endorsement of an item and therefore most closely approximate the affirmative-counting logic of the original CHAEA. This rationale is semantic rather than independently validated psychometrically. Consequently, the binary transformation is treated as a study-specific scoring rule rather than as a validated conversion of the original instrument. A complete respondent who selected only values from 1 to 3 receives a score of zero under this rule; such a record represents low endorsement, not missingness. In particular, the two records containing “Sometimes” responses across all 80 items were retained as complete response patterns.

For participant *i* and item *j*, the administered response $x_{ij}\in \left\{1,2,3,4,5\right\}$ was converted to a binary indicator as

(1) $$b_{ij}=\left\{\begin{array}{cc} 1, & x_{ij}\in \left\{4,5\right\},\\ 0, & x_{ij}\in \left\{1,2,3\right\}. \end{array}\right.$$

For profile *p*, with its 20-item index set $J_{p}$, the participant-level score was

(2) $$S_{ip}=\sum_{j\in J_{p}}b_{ij},\;0\leq S_{ip}\leq 20.$$

The study-specific composite scores were defined as

(3) $$C_{i,\mathrm{analytic}}=\frac{S_{i,\mathrm{Reflective}}+S_{i,\mathrm{Theoretical}}}{2},\;C_{i,\mathrm{action}}=\frac{S_{i,\mathrm{Active}}+S_{i,\mathrm{Pragmatic}}}{2}.$$

These transformations document the analytical scoring procedure but do not imply an established two-factor CHAEA model.

For descriptive purposes, a dominant profile was defined as the profile with the highest binary sum for a participant. Exact ties were retained as combined categories rather than broken arbitrarily. Dominant-profile counts are reported descriptively and are not used as predictors, diagnoses, or treatment groups.

Because the primary 4–5 threshold has not been independently validated, scoring-rule robustness was also examined by repeating the main profile comparisons under alternative representations: binary endorsement at ≥3, the primary rule at ≥4, strict endorsement at =5, and profile means calculated from the original 1–5 responses. These analyses were used as sensitivity checks rather than as evidence of construct validation.

### 3.4. Quantitative Analysis

Data quality checks examined missingness, duplicate identifiers, response ranges, and invariant or near-invariant patterns. A low-variance flag was assigned when at least 90% of a participant’s 80 responses used the same scale point. Four records met this criterion, including two records in which all 80 responses were “Sometimes.” These records were retained in the primary analysis because they were complete and could represent genuine response behavior; all principal tests were then repeated after their exclusion. This low-variance criterion was treated separately from missing-data handling: a complete response pattern was not classified as nonresponse merely because it showed little or no variation.

Profile distributions were summarized with means, standard deviations, medians, interquartile ranges, and observed minima and maxima. Internal consistency for the original five-category items was estimated using coefficient alpha following Cronbach (1951). For the dichotomized items, coefficient alpha is algebraically equivalent to the Kuder–Richardson 20 coefficient under the corresponding binary-item formulation (Cronbach, 1951; Kuder & Richardson, 1937). For a profile with k=20 items, coefficient alpha was calculated as

(4) $$\alpha =\frac{k}{k-1}\left(1-\frac{\sum_{j=1}^{k}\sigma_{j}^{2}}{\sigma_{T}^{2}}\right),$$

where $\sigma_{j}^{2}$ is the variance of item *j* and $\sigma_{T}^{2}$ is the variance of the profile total. Reliability coefficients were interpreted as properties of the scores obtained in the present sample rather than as evidence validating the underlying construct model (McNeish, 2018).

Because the four profile scores were obtained from the same students and were bounded counts with non-normal distributions, an omnibus Friedman test was used (Friedman, 1937). Kendall’s coefficient of concordance, originally developed by Kendall and Babington Smith (1939), was obtained from the Friedman statistic using the relationship described by Legendre (2005):

(5) $$W=\frac{\chi_{F}^{2}}{N(k-1)},$$

where $\chi_{F}^{2}$ is the Friedman statistic, *N* is the number of participants, and k=4 is the number of profiles.

When the omnibus result was significant, all six pairwise contrasts were evaluated with two-sided Wilcoxon signed-rank tests (Wilcoxon, 1945). Exact zero differences were omitted from each contrast, and the resulting *p* values were adjusted across the six comparisons using Holm’s sequentially rejective procedure (Holm, 1979). The matched-pairs rank-biserial correlation was calculated following Kerby (2014) as

(6) $$r_{rb}=\frac{W^{+}-W^{-}}{W^{+}+W^{-}},$$

where $W^{+}$ and $W^{-}$ are the positive and negative signed-rank sums, respectively. The sign indicates the direction of the first profile minus the second. Absolute magnitudes were interpreted comparatively across contrasts and together with the observed score distributions rather than against universal cutoff values.

Scoring-rule sensitivity was examined separately from the low-variance analysis. The four profiles were compared using binary endorsement at ≥3, the primary rule at ≥4, strict endorsement at =5, and means of the original 1–5 responses. For each representation, we repeated the Friedman test, the six paired Wilcoxon contrasts, and the analytic–action-oriented comparison, and counted exact ties in the maximum profile score. Holm adjustment was applied separately to the six pairwise contrasts within each representation and sample. Wilcoxon tests used average ranks, omitted zero differences, and normal approximations without continuity correction. Integer sums and scaled differences were used for rank calculations to preserve exact ties in the original-scale means. Each analysis was repeated after excluding the four low-variance records. These comparisons assessed sensitivity to scoring, not construct validity.

Three exploratory sets of demographic analyses were conducted to determine whether the four profile scores showed substantial variation across variables available for nearly all participants. Female and male students were compared with Mann–Whitney tests and Cliff’s delta; no participant selected another gender response category in the source data. Scores across the six academic programs were compared with Kruskal–Wallis tests. Associations with age were examined using Spearman correlations. Holm adjustment was applied separately within each family of four profile tests. These analyses were explicitly exploratory and were not used to redefine the central interpretation. Mann–Whitney *p* values were obtained using the normal approximation without continuity correction.

Data management, statistical analyses, and data-based figures were produced in R version 4.6.0 (R Core Team, 2026). The workflow used the packages readxl, dplyr, tidyr, stringr, purrr, ggplot2, forcats, scales, and jsonlite. The reproducible analysis workflow generated the score construction, tables, inferential tests, effect sizes, sensitivity analyses, and figures reported in the study.

### 3.5. Qualitative Visual Analysis

The visual strand followed a category-based interpretive analysis informed by thematic-analysis and visual-narrative principles (Braun & Clarke, 2006; Cohn, 2013; Nowell et al., 2017). The unit of analysis was the complete drawing rather than an isolated graphic mark. The analysis began with close description of visible elements—including paths, arrows, stairs, circular forms, barriers, human figures, heads, books, formulas, and directional orientation—before moving to interpretive categories.

Category development combined deductive and inductive procedures. Concepts from the theoretical framework—such as direction, sequence, obstacles, cyclical organization, internal processing, and the presence or absence of social agents—provided initial sensitizing categories, while repeated inspection of the drawings was used to refine category wording and boundaries.

Six nonexclusive summary categories were retained: (1) spatial journey or direction, (2) uncertainty or obstacles, (3) cyclical or radial process, (4) progression by levels, (5) internal cognitive processing, and (6) absence of social agents. A drawing could contribute to more than one category, so the frequencies are descriptive counts rather than mutually exclusive classifications.

Ambiguous or borderline cases were assigned to a category only when the relevant visible feature could be identified without relying on an unavailable participant explanation. When that criterion was not met, the feature was not counted. The available coding summary did not preserve a complete case-level audit trail, and the complete raw corpus was not available for independent recoding; consequently, formal interrater reliability could not be reconstructed.

Interpretive claims were kept close to visible form. Emotional or psychological states were not inferred from the drawings, and written fields that were blank, illegible, or incompletely preserved were excluded from substantive interpretation. To protect participant privacy, original drawings containing handwriting or contextual details that could allow participant re-identification are not reproduced in the article. Instead, recurrent visual forms are illustrated through researcher-generated analytic schematics.

### 3.6. Mixed-Methods Integration

A joint display was used to place aggregate questionnaire results alongside the retained visual patterns (Fetters et al., 2013; Guetterman et al., 2015). Comparisons were organized around orientation, structure, agency, challenge, and social context. These topics provided a framework for describing similarities, differences, and features represented in only one source; they were not treated as shared measured constructs.

No participant-level association or cross-source agreement was estimated. The absence of reliable case linkage, participant explanations, and complete coding records restricted integration to an exploratory comparison of the available summaries. Visual motifs were not used to corroborate preference profiles, infer metacognitive performance, or establish convergence on a common psychological attribute.

## 4. Results

The results are presented in the sequence used for integration: sample characteristics, quantitative profile structure, sensitivity and exploratory analyses, visual narrative patterns, and the mixed-method joint display. Because the qualitative and quantitative records could not be linked case by case, all cross-strand interpretations remain at cohort level.

### 4.1. Participant Characteristics

**Figure 1:**
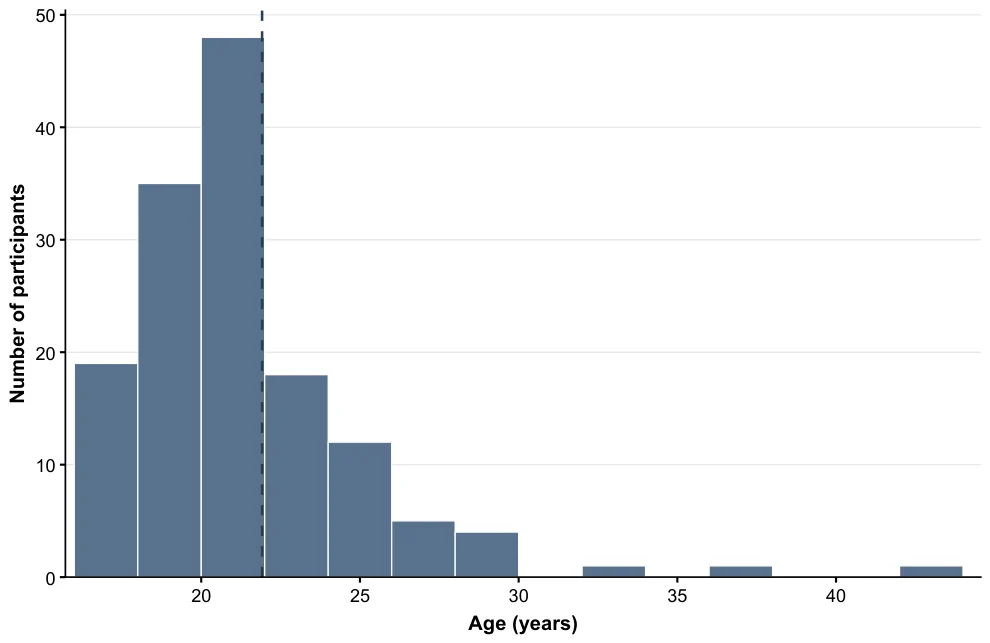
Age distribution of the quantitative sample (N=144). The dashed line marks the sample mean.

The 144 participants had a mean age of 21.92 years (SD=3.73), a median of 22, and a range from 17 to 43. As shown in Figure 1, most students were between 18 and 25, with a modest right tail produced by a small number of older students. The sample included 83 men (57.6%) and 61 women (42.4%). Statistics and Mechatronics Engineering were the largest programs, but all six campus programs were represented. Two thirds of participants reported socioeconomic stratum 1, approximately one fifth were employed, and 30.6% had entered through the Special Admission Program (PAES).

Table 1 provides the detailed demographic and academic characteristics of the quantitative sample.

### 4.2. Learning Preference Score Distributions and Reliability

**Figure 2:**
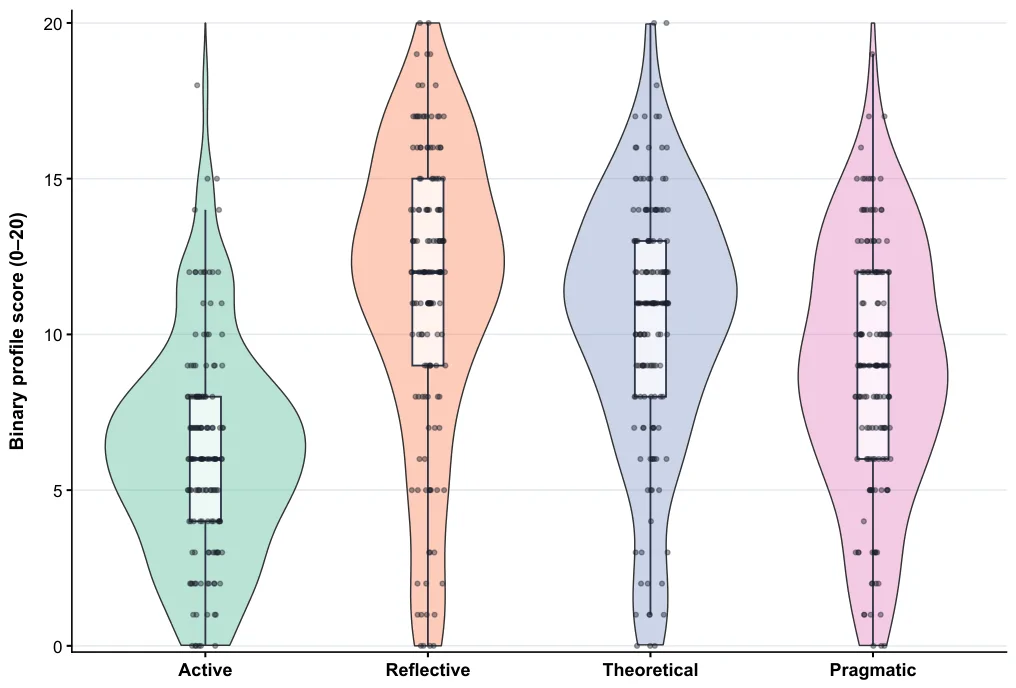
Distributions of the four binary CHAEA-derived profile scores. Note: Each score ranges from 0 to 20. Points represent students; boxes show the median and interquartile range.

Reflective scores were highest (M=11.37, SD=4.79), followed by Theoretical (M=10.37, SD=4.22), Pragmatic (M=8.85, SD=4.08), and Active scores (M=6.24, SD=3.48). Figure 2 shows that the distributions overlapped substantially; the result is a difference in relative location, not a division into nonoverlapping student types. Binary-item reliability ranged from 0.721 for Active to 0.838 for Reflective. Alpha values calculated from the original five-category responses ranged from 0.752 to 0.862 and were higher than the binary-item coefficients for Active, Reflective, and Theoretical, but slightly lower for Pragmatic (0.765 versus 0.770).

Table 2 summarizes the descriptive statistics and internal-consistency coefficients for the four profiles. These coefficients describe consistency among the assigned items in this sample; they do not establish the construct validity of the adapted scores.

### 4.3. Within-Student Profile Contrasts

The Friedman test indicated that the four scores differed within students, $\chi^{2}\left(3\right)=158.27$, p<0.001, with Kendall’s W=0.366. Every pairwise Wilcoxon contrast remained significant after Holm adjustment, as summarized in Table 3. Reflective exceeded Theoretical by a median of one point, a moderate contrast ($r_{rb}=0.410$). Both Reflective and Theoretical exceeded Pragmatic, and all three exceeded Active. The largest difference was observed for the Active–Reflective contrast, for which Active minus Reflective had a median difference of −5 ($r_{rb}=-0.912$), indicating substantially higher Reflective scores.

**Figure 3:**
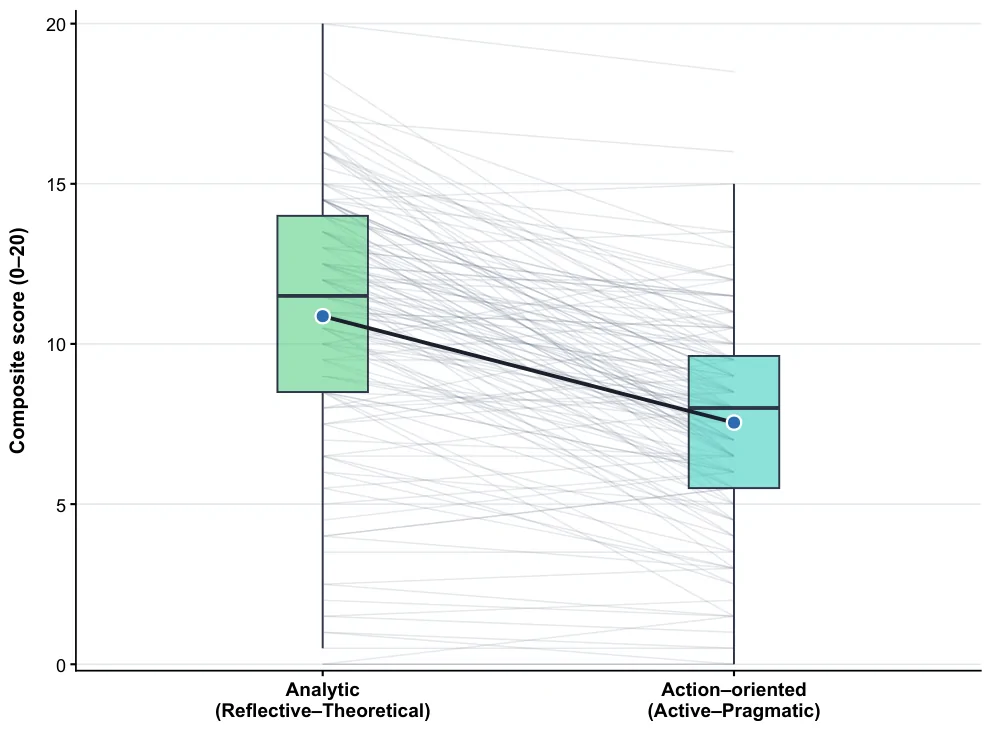
Paired comparison of analytic (Reflective–Theoretical) and action–oriented (Active–Pragmatic) composite scores. Note: Thin lines join the two values for each participant; boxes summarize each distribution.

The analytic composite had a mean of 10.87 (SD=4.30), compared with 7.55 (SD=3.40) for the action-oriented composite. The difference distribution departed modestly from normality, Shapiro–Wilk *W* = 0.9798, p=0.0317. A Wilcoxon signed-rank test confirmed a higher analytic composite, positive-rank V=8935.5, p<0.001, with a large paired rank-biserial correlation, $r_{rb}$ = 0.891. Figure 3 shows the paired distributions. Although the distributions overlapped, the paired comparison indicated that the analytic composite was higher for most individual students.

Removing the four low-variance response patterns did not weaken the conclusion. In the sensitivity sample (n=140), the Friedman result was $\chi^{2}\left(3\right)=161.22$, p<0.001, Kendall’s W=0.384, and the analytic–action contrast remained large ($r_{rb}=0.894$, p<0.001). The primary pattern was therefore not driven by the two all-midpoint records or the other near-invariant responses.

The scoring-rule analyses yielded the same cohort-level ordering under all four representations: Reflective, Theoretical, Pragmatic, and Active. Friedman statistics ranged from $\chi^{2}\left(3\right)=86.32$ to 168.82, with p<0.001 throughout and Kendall’s *W* ranging from 0.200 to 0.391. The analytic composite remained higher than the action-oriented composite ($r_{rb}=0.782$–0.906; all p<0.001). All six pairwise contrasts remained significant after Holm adjustment within each representation; the largest adjusted *p* value was 0.0097 for Reflective versus Theoretical under the strict =5 rule.

The magnitude of profile differentiation and the number of tied maxima nevertheless depended on scoring. Exact ties occurred in 45 records (31.3%) under ≥3, 27 (18.8%) under ≥4, 47 (32.6%) under =5, and 18 (12.5%) when the original responses were retained as profile means. After exclusion of the four low-variance records (n=140), the ordering and the direction of the composite contrast were unchanged across all four representations. Detailed results are provided in Appendix A.3.

### 4.4. Dominant Profiles and Exploratory Demographic Results

**Figure 4:**
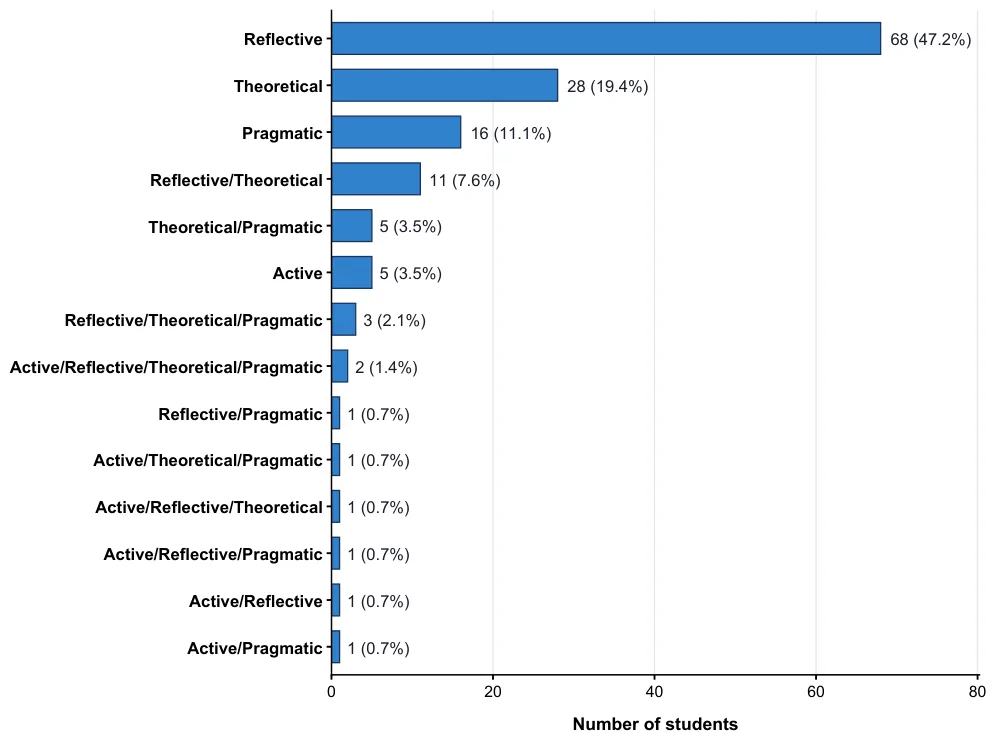
Dominant profile categories based on the highest binary score. Slash-separated labels indicate exact ties.

A single Reflective maximum occurred for 68 students (47.2%), a single Theoretical maximum for 28 (19.4%), a single Pragmatic maximum for 16 (11.1%), and a single Active maximum for five (3.5%). Twenty-seven students (18.8%) had an exact tie involving two or more profiles, including 11 Reflective/Theoretical ties. Figure 4 displays all categories. The frequency of ties reinforces the decision not to treat the four labels as mutually exclusive types.

Exploratory demographic tests found no Holm-adjusted differences by gender or academic program and no adjusted association with age (Appendix A.2, Table A2). The smallest unadjusted program-level *p* value was observed for Theoretical scores (p=0.0467), but it did not remain significant after correction ($p_{Holm}=0.187$). Gender effect sizes were small (absolute Cliff’s δ≤ 0.102), and age correlations were near zero (absolute ρ≤0.077). The central within-student profile pattern was therefore more pronounced than the observed between-group variation.

### 4.5. Visual Narrative Patterns

The following results refer only to the 12 interpretable drawings retained in the visual corpus and should not be generalized to the 30 students originally invited to the qualitative activity. Because the drawings could not be linked reliably to individual questionnaire records, the visual frequencies reported here are not associated with particular learning preference profiles.

All 12 interpretable drawings omitted other learners, teachers, classrooms, or explicit collaborative relationships. Ten drawings (83.3%) used a path, arrow, ascent, or other directional arrangement. Four included visible obstacles, uncertainty, or maze-like structures. Cyclical or radial organization, staged progression, and internal cognitive processing each appeared in approximately three drawings. Table 4 summarizes these nonexclusive categories; because the categories overlapped, percentages do not sum to 100.

**Figure 5:**
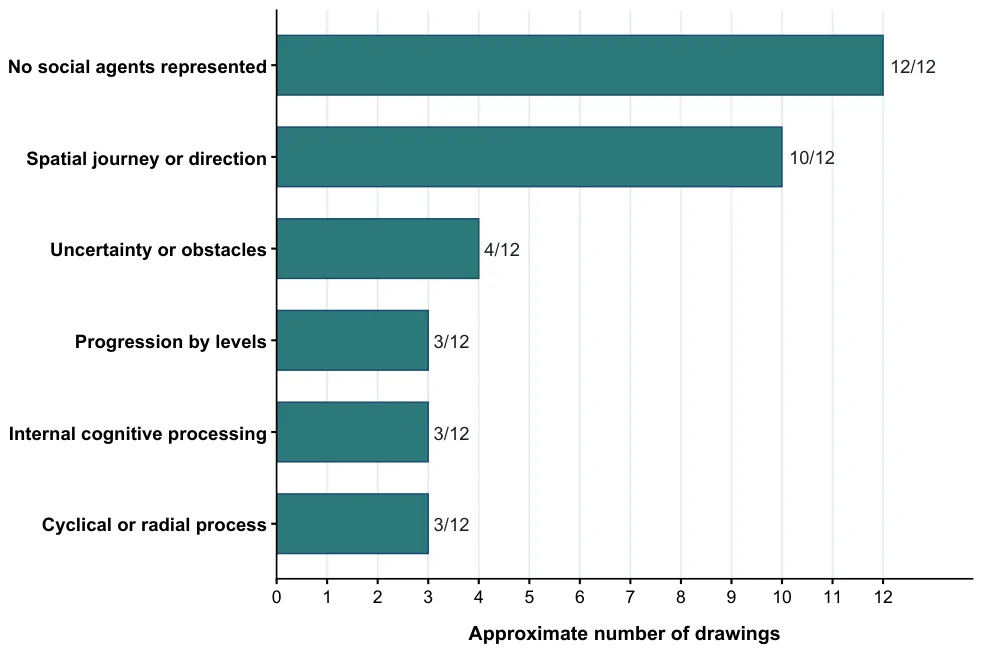
Approximate occurrence of visual narrative motifs in the audited corpus. Categories were nonexclusive.

Figure 5 provides a graphical summary of the frequencies of the six visual categories.

**Figure 6:**
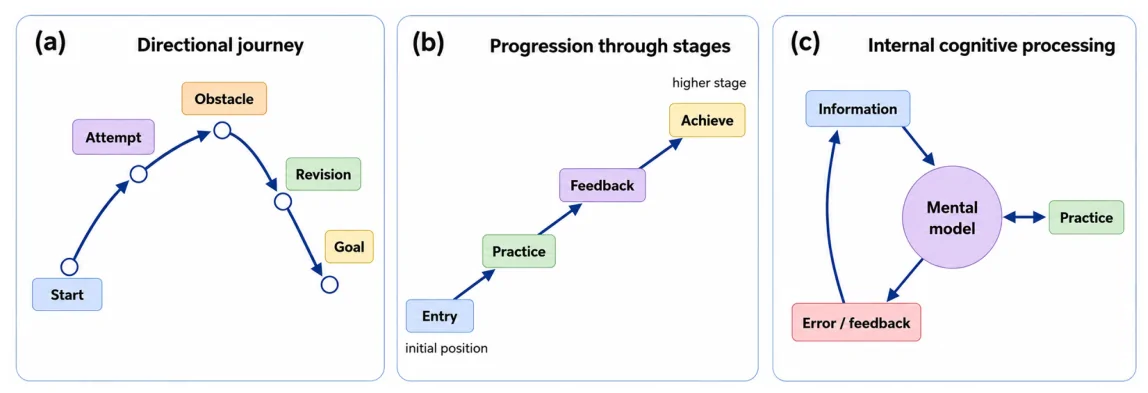
Researcher-generated analytic schematics of three recurrent visual forms: (**a**) directional journey, (**b**) progression through stages, and (**c**) internal cognitive processing.

The visible material most often organized learning as movement toward a destination, sometimes through stages or around obstacles. Internal-processing forms located learning within a system connecting information, thought, and practice. Across the retained corpus, upward or outward direction often coexisted with barriers or discontinuity. These recurring formal features describe how learning was visually represented in the available drawings; without participant explanations, they cannot establish what the images meant to their makers. To illustrate these recurrent forms without reproducing participant material, Figure 6 presents researcher-generated analytic schematics of a directional journey, progression through stages, and internal cognitive processing.

### 4.6. Mixed-Methods Joint Display

Table 5 places the aggregate questionnaire findings alongside the retained visual patterns. Reflective and Theoretical scores were comparatively high, whereas the drawings included directional paths, staged progression, and internally organized forms. These observations describe different sources and do not establish that the visual motifs express the questionnaire dimensions. Movement and practice also appeared in drawings despite lower Active scores, but this contrast cannot be interpreted as agreement or contradiction because the sources do not measure the same behavior and cannot be linked at participant level.

## 5. Discussion

This study described learning preference profiles and visual representations of learning without treating either source as a fixed account of student identity. Quantitatively, Reflective and Theoretical scores were higher than Pragmatic and Active scores, and the analytic composite exceeded the action-oriented composite for most participants. Qualitatively, learning was repeatedly represented as movement through space, progression across stages, or internal processing, while social agents were absent from the retained drawings. At the cohort level, the questionnaire findings emphasized reflection and conceptual structure, whereas the 12 retained drawings provided a narrower visual account of direction, stages, and internal processing. They do not demonstrate that students learn best through reflection alone, that profile scores caused the drawings, or that instruction should be matched to a label.

The connection with human intelligence research is methodological and theoretical. Within this cohort, the ordering of preference scores persisted across four scoring rules, while effect sizes and maximum-profile categories changed. This result supports a descriptive conclusion about the questionnaire responses, not validation of the psychological attributes assigned to them. Similarly, the drawings provide a record of how learning was visually represented, not estimates of monitoring accuracy or regulatory effectiveness. Research on metacognition and self-regulated learning provides a basis for investigating whether such descriptive sources relate to independently measured cognitive and regulatory processes (Dörrenbächer-Ulrich et al., 2024; He et al., 2024). Those relationships were not tested here. The study’s relevance lies in making the distinction between observed patterns and proposed cognitive interpretations explicit and in identifying what additional evidence would be needed to connect them.

### 5.1. Interpretation of the Profile Pattern

The magnitude of the within-student differences deserves attention (Figure 2; Table 2 and Table 3). Kendall’s W=0.366 indicates that the four ranks were not interchangeable, and the analytic–action contrast had a large paired rank-biserial effect. Excluding four low-variance response patterns slightly strengthened rather than weakened the result. The pattern was also more pronounced than any exploratory difference by age, gender, or program. In practical terms, the cohort as a whole reported more frequent behaviors associated with gathering information, considering alternatives, seeking logical structure, and working carefully than behaviors associated with spontaneity, novelty seeking, and rapid experimentation.

The result should not be translated into a claim that nearly half of the sample consists of a distinct kind of “Reflective learner.” The score distributions overlapped, 18.8% of students had exact ties involving two or more profile maxima, and even students with a lower Active score could endorse particular active behaviors. These features are consistent with the criticism that style labels compress a multidimensional and context-sensitive pattern into an apparently stable identity (Newton, 2015; Newton & Miah, 2017). The adapted response format adds another reason for restraint. The internal-consistency estimates do not establish the factor structure, temporal stability, or instructional validity of the adapted categories. The study therefore supports the more limited vocabulary of learning preferences and reported tendencies proposed in recent reconsiderations of the field (Hattie & O’Leary, 2025).

Interpretation of the observed ordering remains conditional on the scoring procedure. The same cohort-level ordering was obtained with all four scoring representations, and the analytic composite consistently exceeded the action-oriented composite. The magnitude of differentiation was not constant, however: Kendall’s *W* was 0.200 under strict endorsement and 0.391 for the original-scale means, while the number of tied maxima ranged from 18 to 47. Thus, the relative ordering was not specific to the primary threshold, but score separation and individual maximum-profile categories remained sensitive to how endorsement was defined. These checks strengthen the evidence for the cohort-level pattern without validating the adapted response format or establishing fixed learner types. Reliability and agreement across scoring rules are not substitutes for construct validation (McNeish, 2018).

This distinction is not merely semantic. When a preference becomes an identity, students may avoid productive difficulty by saying that a task is “not how I learn.” Teachers may also lower expectations by offering only the mode that appears comfortable. The evidence against matching makes that practice difficult to justify (Pashler et al., 2008; Rogowsky et al., 2015). By contrast, a group profile can be useful when it reveals an imbalance in the opportunities students are accustomed to taking. A cohort reporting stronger Reflective and Theoretical tendencies may find conceptual organization and preparation time familiar, but the present study did not test whether particular instructional arrangements would improve learning for these students. Instructional design can instead create opportunities to act before certainty is complete, test provisional models, receive feedback, and revise. The instructional purpose is to expand strategic range, not to protect a preference.

The lower Active score should therefore not be read as a deficit. It could reflect the wording of items that associate activity with spontaneity, risk, sociability, or acting without considering consequences, characteristics that are not synonymous with cognitively active learning. Many evidence-based active-learning tasks are highly structured: students retrieve information, explain a choice, compare solutions, analyze a case, or apply a principle under guidance. Such activity can be compatible with reflective and theoretical orientations because it makes reasoning visible and creates material for subsequent analysis. The broad benefits reported in active-learning research arise from what students think and do with content, not from whether they identify as Active (Dunlosky et al., 2013; Freeman et al., 2014).

The dominant-profile counts also clarify why a single composite comparison was insufficient on its own. Reflective exceeded Theoretical only moderately, while both were much higher than Active. Pragmatic occupied an intermediate position. This ordered pattern suggests that the cohort cannot be summarized as simply “high” versus “low.” Students reported a strong inclination toward analysis, a meaningful orientation toward application, and less frequent endorsement of spontaneity-centered action. These differences may inform hypotheses about how instructional sequences could be structured, but they do not establish an optimal sequence for this cohort. A course might, for example, begin with structure and evidence, move to application through a case or problem, and then increase the openness of experimentation as students gain a conceptual foothold (Fiorella & Mayer, 2016).

### 5.2. Visual Representations of Journey, Structure, and Internal Processing

Interpretation of the visual findings must remain proportional to the available evidence. The qualitative corpus consisted of 12 interpretable drawings, without preserved participant explanations or reliable case-level links to questionnaire scores. The discussion below therefore concerns recurrent formal features in those drawings rather than the prevalence of particular meanings among the 30 students originally invited to the activity.

The visual findings add dimensions that a ranked score cannot supply (Table 4; Figure 5 and Figure 6). Direction was the most common formal feature: learning moved toward a goal, upward through stages, outward from a center, or along a difficult path. These arrangements are consistent with the narrative idea that experience can be organized through beginnings, obstacles, transitions, and possible outcomes (Bruner, 1991; Polkinghorne, 1995). Even drawings without a conventional story contained an implied temporality. A staircase presupposes a before and after; a maze places the learner inside uncertainty; and a radial figure presents understanding as accumulation or expansion.

The images also resisted a single model of progress. Some were linear and hierarchical, while others were cyclical, branching, or discontinuous. This contrast matters because a staircase can make orderly advancement visible, whereas a maze can represent false starts, ambiguity, and revision. These forms should not be treated as transparent statements of students’ beliefs or internal states, because visual meaning depends on representational conventions and the elicitation context (Cohn, 2013; Quillin & Thomas, 2015). They are observable representational features whose meanings cannot be established confidently without participants’ explanations. Together, however, they show that the retained corpus included both structured progression and discontinuity.

The recurrence of visual abstraction is consistent with research on drawing as a means of selecting and organizing relations (Ainsworth et al., 2011; Quillin & Thomas, 2015). The drawings were generally schematic rather than realistic: arrows, levels, geometric forms, and cognitive icons carried much of their organization. Such economy should not automatically be interpreted as lack of meaning, since a simple configuration can function as a model when the prompt concerns a broad concept such as learning. At the same time, the images cannot be interpreted independently of the elicitation context. Without participant explanations, the analyst cannot know whether a spiral represented repetition, growth, confusion, or another idea. Interpretation therefore remains anchored in recurring visible relations.

The absence of teachers, peers, classrooms, and explicit collaborative relationships was the most consistent formal feature across the 12 retained drawings (Table 4). This finding is limited to what was represented in the available images. It does not establish that students learn in isolation, prefer autonomous learning, avoid help seeking, or perceive social support as unimportant. The individual framing of the prompt, representational conventions, and other contextual conditions may influence what participants choose to include in a visual account (Farrar et al., 2024; Jewitt, 2008).

Learning is socially organized even when cognitive work feels private. Instructors select problems, sequence explanations, model standards, create feedback loops, and establish whether uncertainty can be expressed safely. Peers can provide alternative interpretations and expose assumptions. The absence of these agents from the retained images therefore raises a question for future research rather than demonstrating a deficit in students’ learning environments. Future elicitation could explicitly ask participants who or what supports learning and could combine drawings with brief explanations to distinguish task framing from broader perceptions of social learning.

The sparse, illegible, or incompletely preserved written material is not interpreted as a substantive finding. The available archive does not allow blank responses, uncertainty about the prompt, limited time, reluctance to write, or incomplete preservation to be distinguished reliably. Consequently, the absence of usable written narratives provides no evidence of a visual learning preference or of a multimodal advantage.

### 5.3. Scope of the Mixed-Methods Integration

As Fetters et al. (2013) describe, integration can occur when separately analyzed findings are brought together during interpretation and reporting. In this study, that comparison was limited by the unequal extent and completeness of the two sources. The questionnaire provided 144 complete response profiles, whereas the visual material comprised 12 interpretable drawings without participant explanations, complete coding records, or reliable case linkage.

Guetterman et al. (2015) identify joint displays as a way to organize quantitative and qualitative findings for comparison. Here, Table 5 makes the differences between the available sources explicit. Questionnaire totals summarize item endorsement; visual categories summarize recurring forms in the retained material. The table does not establish that both sources express a shared metacognitive orientation or independently confirm the same finding.

The presence of movement and practice in drawings alongside lower Active scores is therefore not evidence of either convergence or contradiction. The questionnaire items and visual motifs address different aspects of learning-related expression, and their relationship cannot be determined from the available records. A participant-level explanation would require linked observations and participants’ accounts of their drawings.

Similarly, the absence of social agents in the drawings has no direct counterpart in the profile totals. It identifies a feature of the retained visual material, not a demonstrated pattern of collaboration, autonomy, or help seeking in the cohort. Future research could examine these relationships using linked data, preserved coding records, participant explanations, and appropriate measures of the proposed constructs.

### 5.4. Implications for University Teaching

The present study did not implement an instructional intervention or measure learning outcomes. The following implications should therefore be understood as design hypotheses informed by the observed patterns and by prior educational research, not as teaching approaches shown to be effective for this cohort.

One possible instructional architecture could provide multiple entry points within a shared learning sequence. It might begin by orienting students to the problem, purpose, success criteria, and conceptual structure; provide material for careful observation; invite students to generate a representation or explanation; move into application through a case, simulation, problem, field task, or small experiment; and close with comparison, feedback, and reflection. Such a sequence is consistent with generative and active-learning principles (Fiorella & Mayer, 2016; Freeman et al., 2014), but its effectiveness in this setting remains to be tested.

Narrative may provide one way to connect these phases. A disciplinary case can offer an event to enter, evidence to inspect, a model to construct, and a consequence to evaluate. The same narrative can be encountered in several forms rather than duplicated as separate lessons for presumed learner types. Students might read a concise case, sketch the system, identify a turning point, test a solution with data, and write a short post-task explanation. The potential benefit would arise from the cognitive work of selecting, organizing, integrating, and revising rather than from matching instruction to a declared preference (Fiorella & Mayer, 2016).

The visual findings suggest additional questions for instructional design. Teachers might make feedback, peer contribution, consultation, and revision more visible in instructional representations, and they might normalize nonlinear progress by showing how disciplinary understanding develops through error, comparison, and revision. These possibilities follow from features observed in the small visual corpus and should be evaluated prospectively rather than treated as needs demonstrated by the present study.

Digital storytelling and student-generated media can extend these practices, but they should remain aligned with learning goals. Ginting et al. (2024) reported educational benefits when digital storytelling required students to organize content into a meaningful communicative product, consistent with earlier work on narrative-based digital production (Robin, 2008; Yang & Wu, 2012). A technically elaborate video is not automatically more educational than a hand-drawn model. The central design question is what relationships students must notice, explain, and transfer. Low-technology options should remain available so that multimodality does not become a new access barrier.

Generative artificial intelligence makes this principle even more important. Text and image systems can help students brainstorm, compare representations, or receive feedback, but they can also produce fluent artifacts that conceal weak understanding. Current reviews emphasize ethical concerns, unequal access, accuracy, transparency, and the need for human oversight (Bond et al., 2024; Farrokhnia et al., 2024; Yan et al., 2024). In this context, student explanations of why a representation was chosen, what evidence supports it, and how it changed may be more informative than the polish of the final product.

These implications align more closely with inclusive design than with learning-style accommodation. Inclusion asks whether students can enter the task, participate meaningfully, and demonstrate learning through supported routes while still engaging with shared disciplinary goals (González-Afonso et al., 2026). Multiple representations can expand access and strategic flexibility; they should not become separate tracks that confine students to what they already prefer.

### 5.5. Limitations and Directions for Future Research

Several limitations narrow the claims. The quantitative sample came from one regional campus and was not randomly selected from a defined national population. The number of students invited to participate and a defensible response-rate denominator were not preserved in the available documentation. Program sizes were unequal, and unmeasured factors such as semester, prior achievement, first-generation status, language background, or course experience may shape responses. The demographic analyses were exploratory and may have had limited power for subtle differences. Generalization should therefore be analytical—to similar questions and contexts—rather than statistical to Colombian higher education as a whole.

The instrument was adapted from a dichotomous questionnaire to a five-category frequency form. Binarizing responses of 4 and 5 reproduced the source analysis but introduced a threshold that lacks independent validation. The study did not conduct confirmatory factor analysis, test–retest reliability, measurement invariance, or criterion validation against observed learning behavior. As McNeish (2018) emphasizes, internal consistency alone does not establish construct validity. Dominant categories were sensitive to small score differences and should not be used in decisions about individual students. The scoring-rule analyses reproduced the cohort-level ordering, but effect sizes and the frequency of tied maxima varied across representations. Sensitivity checks therefore do not resolve the need to establish the dimensional structure, measurement invariance, and criterion validity of the adapted instrument. Future work should evaluate binary and ordinal measurement models, use preregistered psychometric models, and prioritize strategy or self-regulation measures with stronger links to learning outcomes.

The study measured neither intellectual ability nor metacognitive monitoring and did not evaluate achievement or an instructional intervention. It cannot show that narrative teaching improves comprehension for this cohort, that the analytic profile causes performance, or that matching would fail in this particular setting. A stronger next study could compare instructional sequences that all contain evidence-based learning processes but differ in how narrative and visual representation are integrated. Outcomes should include immediate understanding, delayed retention, transfer, participation, and students’ ability to explain their own strategy use.

The qualitative limitations are more substantial. Only 12 drawings were interpretable in the audited documentation, the complete raw corpus and original coding sheet were unavailable, and participant explanations were not preserved. No participant-level key linked drawings to questionnaire scores. Approximate frequencies therefore describe the archived material rather than a fully recoverable qualitative dataset. Coding was not independently replicated, and the study cannot claim meaning saturation. The available written material also cannot support substantive claims about verbal nonresponse because blank, illegible, and incompletely preserved responses cannot be distinguished reliably. Future data collection should preserve every artifact, record the circumstances and time available, obtain short elicitation interviews, use at least two analysts for a documented subset, and maintain a de-identified linking key.

Finally, visual interpretation is shaped by the prompt and by the analysts’ conceptual vocabulary. A path may carry different meanings for different students, and the absence of people may reflect the instruction to portray “your learning” rather than a genuinely individualistic conception. Member reflection—asking participants whether an interpretation fits, partly fits, or misses their intent—would strengthen credibility. Longitudinal visual narratives could also show whether students’ metaphors change as they enter advanced courses, participate in research, or encounter more collaborative pedagogies.

Within these limits, the principal contribution is a bounded mixed-methods account of what the available evidence supports: a clear group-level ordering of preference scores, a small visual corpus organized around direction, structure, effort, and internal processing, and a cohort-level comparison that does not convert preferences into intelligence measures, drawings into diagnoses, or pedagogical possibilities into demonstrated effects.

## 6. Conclusions

This mixed-methods study found a clear ordering in CHAEA-derived learning preference scores among 144 students at the Universidad Nacional de Colombia, Sede de La Paz. Reflective scores were highest, followed by Theoretical, Pragmatic, and Active scores. The analytic composite exceeded the action-oriented composite with a large paired effect, and this pattern remained stable after low-variance responses were removed and across all four scoring representations. These results describe how the cohort reported approaching learning; they do not establish fixed learner types, validate the adapted instrument for individual diagnosis, or support matching instructional media to a dominant profile. The repeated ordering describes responses under the examined scoring rules, not validated differences in psychological capacities. Neither intellectual ability nor metacognitive performance can be inferred from these scores.

The visual corpus added a different layer of understanding. Across 12 interpretable drawings, learning was usually represented as a journey, an ascent, a sequence of stages, or an internal cognitive system. Obstacles and uncertainty appeared alongside upward or goal-directed movement. Teachers, peers, and classroom settings were not represented in the retained drawings, but this absence should be understood as a feature of the available visual material rather than as evidence that students actually learn in isolation or prefer individual learning. Because participant explanations were not preserved, the meanings assigned to these visual forms cannot be established beyond their observable representational features. The limited visual corpus therefore complements the quantitative findings descriptively but does not support generalization to all students originally invited to the qualitative activity.

The joint display places two types of descriptive evidence alongside each other; it does not establish convergence on a common psychological construct. The findings nevertheless suggest possible directions for instructional design based on sequencing and multimodality rather than labeling. Students may be offered conceptual orientation and opportunities to inspect evidence, construct and explain representations, apply ideas in cases or problems, and reflect on feedback and revision. Narratives may connect these activities within a common task without requiring separate instruction for presumed learner types. These possibilities should be treated as pedagogical hypotheses, not as interventions shown to improve learning in this cohort, because the study did not evaluate achievement or instructional effects.

The principal empirical finding is a consistent ordering of CHAEA-derived scores across four scoring representations, with variation in effect sizes and tied maxima. The small visual corpus provides a separate descriptive record of recurrent forms rather than confirmation of the questionnaire profiles. The relevance to human intelligence research concerns the interpretation of these learning-related sources and the evidence needed to connect them with cognitive and regulatory constructs. Future research should validate the response format, preserve linked multimodal records, obtain participant explanations, and directly assess any proposed relationship with metacognition or intelligence. Instructional applications likewise require prospective evaluation.

## Figures and Tables

**Table 1:** Participant characteristics (N=144).

Characteristic	Value	%
Age, mean (SD), years	21.92 (3.73)	
Age, median (IQR), years	22 (20–23)	
Age range, years	17–43	
Men	83	57.6
Women	61	42.4
Statistics	36	25.0
Mechatronics Engineering	32	22.2
Biology	21	14.6
Biological Engineering	20	13.9
Geography	18	12.5
Cultural and Communication Management	17	11.8
Socioeconomic stratum 1	96	66.7
Socioeconomic stratum 2	36	25.0
Other/no reported stratum	12	8.3
Currently employed	30	20.8
PAES admission route	44	30.6

**Table 2:** Profile score descriptives and internal consistency (N=144).

Profile	*M*	SD	Median	IQR	Min	Max	$\alpha_{\mathrm{binary}}$	$\alpha_{\mathrm{Likert}}$
Active	6.24	3.48	6	4–8	0	18	0.721	0.752
Reflective	11.37	4.79	12	9–15	0	20	0.838	0.862
Theoretical	10.37	4.22	11	8–13	0	20	0.788	0.809
Pragmatic	8.85	4.08	9	6–12	0	19	0.770	0.765

Note. $\alpha_{binary}$ is coefficient alpha/KR-20 for dichotomized items; $\alpha_{Likert}$ uses the original five response categories.

**Table 3:** Pairwise Wilcoxon signed-rank comparisons among profile scores.

Contrast (First Minus Second)	$W_{\min}$	Median Difference	$r_{\mathrm{rb}}$	Holm-Adjusted *p*
Active–Reflective	404.5	−5	−0.912	<0.001
Active–Theoretical	459.5	−4	−0.895	<0.001
Active–Pragmatic	957.0	−2	−0.772	<0.001
Reflective–Theoretical	2176.0	1	0.410	<0.001
Reflective–Pragmatic	1355.0	2	0.696	<0.001
Theoretical–Pragmatic	1451.0	2	0.613	<0.001

Note. $W_{\min}=\min\left(W^{+},W^{-}\right)$ is the smaller of the two rank sums in the Wilcoxon signed-rank test. Here, $W^{+}$ and $W^{-}$ are the sums of the ranks of absolute paired differences associated with positive and negative differences, respectively, using the first-minus-second convention. $r_{rb}$ is the paired rank-biserial correlation. Zero differences were omitted separately for each comparison; Holm correction covered six tests.

**Table 4:** Approximate frequencies of visual narrative categories in the retained corpus (n=12 interpretable drawings).

Category	*n*	%
No social agents represented	12	100.0
Spatial journey or direction	10	83.3
Uncertainty or obstacles	4	33.3
Cyclical or radial process	3	25.0
Progression by levels	3	25.0
Internal cognitive processing	3	25.0

**Table 5:** Joint display of aggregate questionnaire findings and the retained visual material.

Integrative Dimension	Quantitative Pattern	Visual Pattern	Descriptive Comparison
Pace and orientation	Reflective highest; Active lowest	Paths were common; staged progression was also represented	The profile ordering and directional motifs describe different aspects of the available material; neither establishes deliberate regulation.
Structure	Theoretical second-highest; moderate Reflective–Theoretical difference	Levels, radial forms, and cognitive diagrams	Visual organization does not establish that the drawings express the Theoretical profile or corroborate its questionnaire score.
Agency and effort	Action composite lower but present across the range	Obstacles, practice, arrows, and goals	The visual material adds representations of action and effort not captured by the composite comparison; individual correspondence cannot be established.
Sociality	Instrument includes interpersonal items but no social composite	No teachers, peers, or classrooms in the retained drawings	Social agents were not represented in the retained visual corpus; this absence does not establish actual social isolation, autonomy, or help-seeking behavior.

## Data Availability

The anonymized Spanish- and English-language quantitative datasets, variable dictionaries, R analysis script, and README file are openly available in Mendeley Data (Correa, 2026). The complete visual corpus is not publicly deposited because handwriting and contextual features could permit participant re-identification. To protect privacy, participant drawings are not reproduced in the article; the recurrent visual forms are instead illustrated through researcher-generated analytic schematics.

## Appendix A. Supplementary Methodological Information

This appendix provides additional methodological information supporting the transparency and reproducibility of the study without interrupting the flow of the main text. It includes the allocation of the 80 questionnaire items to the four learning preference profiles, the complete results of the exploratory demographic analyses, and the sensitivity of profile comparisons to alternative scoring representations.

### Appendix A.1. Allocation of Questionnaire Items to Learning-Preference Profiles

Table A1 documents the item allocation used to construct the four CHAEA-derived profile scores analyzed in the main text.

**Table A1:** Item numbers assigned to each profile in the 80-item questionnaire.

Profile	Item Numbers
Active	3, 5, 7, 9, 13, 20, 26, 27, 35, 37, 41, 43, 46, 48, 51, 61, 67, 74, 75, 77
Reflective	10, 16, 18, 19, 28, 31, 32, 34, 36, 39, 42, 44, 49, 55, 58, 63, 65, 69, 70, 79
Theoretical	2, 4, 6, 11, 15, 17, 21, 23, 25, 29, 33, 45, 50, 54, 60, 64, 66, 71, 78, 80
Pragmatic	1, 8, 12, 14, 22, 24, 30, 38, 40, 47, 52, 53, 56, 57, 59, 62, 68, 72, 73, 76

### Appendix A.2. Exploratory Demographic Associations

Table A2 summarizes the exploratory associations between the four primary profile scores and gender, academic program, and age.

**Table A2:** Exploratory associations between profile scores and demographic variables.

Analysis	Profile	Test Statistic	Effect/Association Estimate	df	Raw *p*	Holm *p*
Gender (women vs. men)	Active	U=2273.0	δ=−0.102	—	0.295	1.000
	Reflective	U=2376.5	δ=−0.061	—	0.531	1.000
	Theoretical	U=2620.5	δ=0.035	—	0.720	1.000
	Pragmatic	U=2363.5	δ=−0.066	—	0.497	1.000
Academic program (6 groups)	Active	H=6.56	—	5	0.255	0.511
	Reflective	H=9.68	—	5	0.085	0.254
	Theoretical	H=11.25	—	5	0.047	0.187
	Pragmatic	H=5.56	—	5	0.351	0.511
Age (Spearman)	Active	—	ρ=0.067	—	0.423	1.000
	Reflective	—	ρ=−0.077	—	0.360	1.000
	Theoretical	—	ρ=−0.048	—	0.572	1.000
	Pragmatic	—	ρ=−0.0003	—	0.997	1.000

Note. Cliff’s δ is reported for gender comparisons, and Spearman’s ρ is reported for associations with age. For Kruskal–Wallis comparisons across academic programs, df=5. Holm correction was applied separately within each four-test family.

### Appendix A.3. Robustness to Alternative Scoring Representations

Table A3 summarizes the scoring-rule sensitivity analyses for all 144 complete records. Panel A reports the profile means and exact ties in the maximum score; Panel B reports the omnibus and composite comparisons and the largest Holm-adjusted pairwise *p* value under each rule.

**Table A3:** Sensitivity of profile comparisons to alternative scoring representations (N=144).

**Panel A. Profile Means and Tied Maxima**					
**Scoring Rule**	**Active**	**Reflective**	**Theoretical**	**Pragmatic**	**Ties, n (%)**
Binary (≥3)	13.86	17.83	17.20	16.28	45 (31.3%)
Binary (≥4; primary)	6.24	11.37	10.37	8.85	27 (18.8%)
Binary (=5)	1.50	3.65	3.08	2.23	47 (32.6%)
Original 1–5 means	3.01	3.62	3.51	3.32	18 (12.5%)
**Panel B. Within-Student Comparisons**					
**Scoring Rule**	$\chi_{F}^{2}\left(3\right)$	**Kendall’s *W***	$V^{+}$	$r_{\mathrm{rb}}$	**Max. Holm *p***
Binary (≥3)	152.31	0.353	8432.5	0.893	<0.001
Binary (≥4; primary)	158.27	0.366	8935.5	0.891	<0.001
Binary (=5)	86.32	0.200	6575.0	0.782	0.010
Original 1–5 means	168.82	0.391	9406.0	0.906	<0.001
